# Enhancing SiGeSn nanocrystals SWIR photosensing by high passivation in nanocrystalline HfO_2_ matrix

**DOI:** 10.1038/s41598-024-53845-z

**Published:** 2024-02-12

**Authors:** Ioana Dascalescu, Catalin Palade, Adrian Slav, Ionel Stavarache, Ovidiu Cojocaru, Valentin Serban Teodorescu, Valentin-Adrian Maraloiu, Ana-Maria Lepadatu, Magdalena Lidia Ciurea, Toma Stoica

**Affiliations:** 1 National Institute of Materials Physics, 405A Atomistilor Street, 077125 Magurele, Romania; 2 Academy of Romanian Scientists, 54 Splaiul Independentei, 050094 Bucharest, Romania

**Keywords:** Group IV alloys, HfO_2_, SiGeSn nanocrystals, Magnetron sputtering, SWIR, Spectral photocurrent, Nanoscience and technology, Nanoscale materials, Nanoparticles

## Abstract

SiGeSn nanocrystals (NCs) in oxides are of considerable interest for photo-effect applications due to the fine-tuning of the optical bandgap by quantum confinement in NCs. We present a detailed study regarding the silicon germanium tin (SiGeSn) NCs embedded in a nanocrystalline hafnium oxide (HfO_2_) matrix fabricated by using magnetron co-sputtering deposition at room temperature and rapid thermal annealing (RTA). The NCs were formed at temperatures in the range of 500–800 °C. RTA was performed to obtain SiGeSn NCs with surfaces passivated by the embedding HfO_2_ matrix. The formation of NCs and β-Sn segregation were discussed in relation to the deposition and processing conditions by employing HRTEM, XRD and Raman spectroscopy studies. The spectral photosensitivity exhibited up to 2000 nm in short-wavelength infrared (SWIR) depending on the Sn composition was obtained. Comparing to similar results on GeSn NCs in SiO_2_ matrix, the addition of Si offers a better thermal stability of SiGeSn NCs, while the use of HfO_2_ matrix results in better passivation of NCs increasing the SWIR photosensitivity at room temperature. These results suggest that SiGeSn NCs embedded in an HfO_2_ matrix are a promising material for SWIR optoelectronic devices.

## Introduction

SiGeSn semiconductor alloys are a promising material for a broad area of optoelectronics applications compatible with silicon technology. The SiGeSn ternary alloy presents two main advantages compared to the binary alloys of SiGe and GeSn, namely: (i) the electronic band structure can be modified without varying the lattice mismatch strain constant, by tuning Si and Sn content; (ii) the thermal stability of SiGeSn is increased compared to GeSn due to the local strain compensation induced by Si-Sn atomic pairs in the Ge lattice^1^.

The research on nanostructures based on (Si)GeSn epitaxial films, heterojunctions, nanowires and nanocrystals (NCs) represents a hot-topic field from fundamentals to applications^2–7^. The alloying of Ge with Sn is obtained by controlling non-equilibrium growth kinetics of GeSn thin films, the thermal solubility of Sn in Si and Ge being limited at less than 1%. By alloying Ge with Sn, the bandgap has a transition from the indirect Ge bandgap to the direct bandgap, by increasing the Sn concentration above 6–8%, thereby increasing the band edge optical absorption and light emission efficiency, making the group IV alloy semiconductor suitable for optoelectronic/photonic applications^8^. More than that, this effect leads to the increase of the laser emission efficiency in SWIR^9^. In addition, a decrease in bandgap is achieved down to the 3 µm wavelength limit in SWIR and even further in the Mid-IR range, GeSn thus extending the SWIR photosensitivity of Ge^10,11^. On the other hand, adding Si to GeSn helps reducing the local stress field around Sn atoms and increases the alloy thermal stability, that is associated with the increased mixing entropy in ternary SiGeSn^12^.

In the case of epitaxially grown GeSn layers on Ge buffered Si substrates, the thermal stability is limited by plastic relaxation of the strain through dislocations, as well as by Sn segregation. It was shown by post deposition annealing of epi-GeSn (5–12% Sn) that in pseudomorphic GeSn layers grown by chemical vapor deposition (CVD) the Sn segregation progressively leads to the accumulation of Sn at the film surface, while in partially relaxed, in addition to a further plastic relaxation, there is at a critical temperature a sudden massive Sn segregation mediated by dislocations and interface defects^13^. Sn segregation in epitaxial GeSn by formation of Sn-rich clusters was also reported for GeSn films grown by molecular beam epitaxy (MBE), very likely induced by local structural defects^14^. The critical temperature decreases with Sn content and is about of 400 °C for a concentration of 12% Sn. EXAFS measurements on SiGeSn layers showed that the addition of Si to GeSn effectively increases the thermal stability by reducing the local stress and increasing the Sn segregation temperature^15^.

The self-formation of GeSn NCs in oxide was obtained by annealing of GeSnSiO_2_ alloy films deposited by magnetron sputtering (MS) of Ge, Sn and SiO_2_ from separate targets.^6,7^ Sn and Ge segregate from GeSnSiO_2_ alloy at 400 °C to form GeSn NCs of cubic α-structure, that of Ge. Increasing the annealing temperature, GeSn nanocrystallization progressively increases and segregation of metallic β-Sn already occurs at 450 °C. The SiO_2_ matrix plays an important role in NCs formation and NCs boundary passivation. Thus, without or for low SiO_2_ concentration alloying, nanocrystallization of amorphous GeSn and β-Sn segregation take place at lower temperatures 300–350 °C and the SWIR photosensitivity is dramatically diminished^7^. On the other hand, the Ge nanocristallization in GeSiO_2_ alloys without Sn addition occurs at clearly higher temperatures of 600–650 °C^16,17^. On contrary, the Si substantial addition increases the thermal stability by increasing the nanocrystallization temperature, e.g. for GeSi NCs at 900 °C^24^ in comparison to Ge NCs at 600 °C, while the nanocrystallization temperature for GeSn is increased by Si addition from 400 °C^6,7^ to 520–530 °C in Ref.^26^. The nature of the oxide matrix can also influence the nanocrystallization of SiGeSn alloys, as shown in present paper for the case of HfO_2_ in which the nanocrystallization of the matrix can serve as a support for the nucleation of the cubic SiGeSn phase, helping the segregation and formation of SiGeSn NCs.

In optoelectronic applications^18^, the concentration of Sn in the layer plays a crucial role in determining the material performance^19^. A high percentage of Sn affects the material photoresponse by reducing the quantum efficiency. However, an optimal concentration of Sn is required to achieve the desired performance. By adding Si to GeSn the bandgap increase, but the photosensitivity threshold still can remain in SWIR range. It is also worth mentioning that this ternary alloy can switch from an indirect bandgap to a direct bandgap if the concentration of Si is less than 4% and the concentration of Sn is greater than 9%.

In addition to the added complexity of using a ternary SiGeSn alloy instead of the binary SiGe and GeSn alloy that allow the band gap and strain values to be adjusted within wide limits, the choice of the HfO_2_ matrix instead of the SiO_2_ has the role of electronic confinement effects in SiGeSn NC by using a high dielectric constant oxide. Both group-IV SiGeSn and HfO_2_ are CMOS compatible, HfO_2_ being standard in micro- and nanoelectronics and highly scalable, extending the capabilities of Si and HfO_2_ for optoelectronic applications.

SiGeSn films of high crystalline quality are usually obtained by CVD^20,21^ and MBE on Ge buffered Si^22,23^. Epitaxial growth of SiGeSn films by MS was also demonstrated^24^. However, the obtaining of high-quality epitaxial films of high content of Sn is still challenging due to formation of dislocations and segregation of Sn. Due to their non-toxic nature and compatibility with Si technology, SiGeSn-based photodetectors represent a recommended alternative to the commonly available III–V devices.

The use of films of (Si)GeSn NCs embedded in oxide matrix obtained by MS deposition for optical sensors represents a cost-effective fabrication solution. The oxide matrix in these composite materials plays a crucial role in passivating the NCs surface, essential for their photoelectrical performance^25^. This technique was successfully developed for Ge, GeSi and GeSn NCs embedded in different oxides, for photosensing applications^5–7,25–29^.

In this work, we present the formation and SWIR optoelectric properties of SiGeSn NCs embedded in HfO_2_ matrix. Layers of SiGeSnHfO_2_ were obtained by magneton co-sputtering of SiGe, Sn, and HfO_2_. The nanocrystallization was obtained by rapid thermal annealing (RTA) at temperatures in the range of 500–800 ℃, the deposition being performed on Si substrates maintained at room temperature. The high-quality NC/matrix interface allows to fully exploit quantum confinement effect and high SWIR sensitivity. The formation of SiGeSn NCs and β-Sn segregation are discussed, in relation to the deposition and processing conditions. Depending on the Sn content of SiGeSn NCs, the spectral photocurrent exhibits a different SWIR sensitivity. Extended sensitivity up to 2000 nm was measured for SiGeSn NCs with about 8 at.% Sn composition.

## Results and discussion

SiGeSnHfO_2_ films on p-Si substrates were obtained by MS co-deposition using three separate targets (SiGe 50 at.% alloying, pure Sn and HfO_2_ targets—see “Methods” section). The samples annealed by RTA at different temperatures have been investigated by HRTEM, XRD, μ-Raman and photocurrent measurements.

### Structure, morphology and composition studies

#### HRTEM analysis

**Figure 1:**
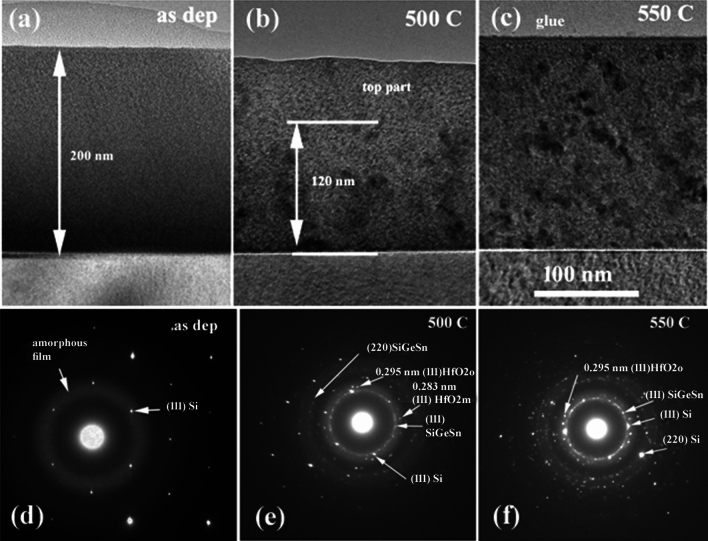
Image showing the cross-section morphology of the film in the as-dep state (**a**) and after annealing at (**b**) 500 °C and (**c**) 550 °C; SAED patterns of the crystallized structure (**d–f**). The selected area for SAED patterns has a part of Si substrate, that gives Si (111) reflection points additionally to the amorphous ring. The figure (**b**) shows only a part of the total thickness of the film, due to selected zone of the TEM specimen.

The as-deposited (as-dep) film has a thickness of 200 nm and shows a uniform amorphous structure as shown by TEM image in Fig. 1a. After RTA at 500 °C, the film presents a beginning of crystallization, mainly in the bottom part of the film, close to the Si substrate (Fig. 1b). RTA at 550 °C leads to almost total crystallization of the film (Fig. 1c). The corresponding crystallization states for different annealing states are well revealed by the selected area electron diffraction (SAED) images shown in Fig. 1d–f. In the electron diffraction patterns, one can see that the amorphous ring is still present in RTA 500 °C sample (Fig. 1e), but it is less pronounced for 550 °C RTA (Fig. 1f). The reflections rings of (111) and (220) planes of the cubic structure of the SiGeSn alloy are present in the SAED patterns after annealing at 500 °Cand 550 °C (Fig. 1e, f). Some other spots belonging to the HfO_2_ nanocrystallites also appear.

**Figure 2:**
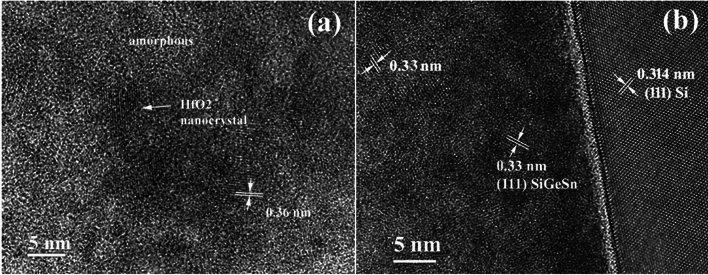
HRTEM image of the interface area of the 500 °C sample: (**a**) detail in the middle part of the film thickness; (**b**) the bottom part of the film at the interface with the Si substrate.

Only a small part of the film is crystallized in the middle part of the film after 500 °C RTA (Fig. 2a). By counting the number of the lattice fringes observed in the HRTEM image (Fig. 2a), we have found crystallites of 5 to 10 nm sizes, surrounded by an amorphous matrix. The presence of the lattice fringes spaced at 0.36 nm indicates the HfO_2_ crystallization.

Figure 2b shows the high-resolution structure at the interface area. Most of the crystallites that form in the amorphous matrix with a size of about 5 nm belong to the cubic structure of the SiGeSn alloy. These can be observed and measured by lattice fringes of 0.33 nm that are specific to the sequence of planes (111) of the cubic structure of SiGeSn. Some larger crystallites (10 nm), better crystallized and originating from the HfO_2_ type structure appear less often.

**Figure 3:**
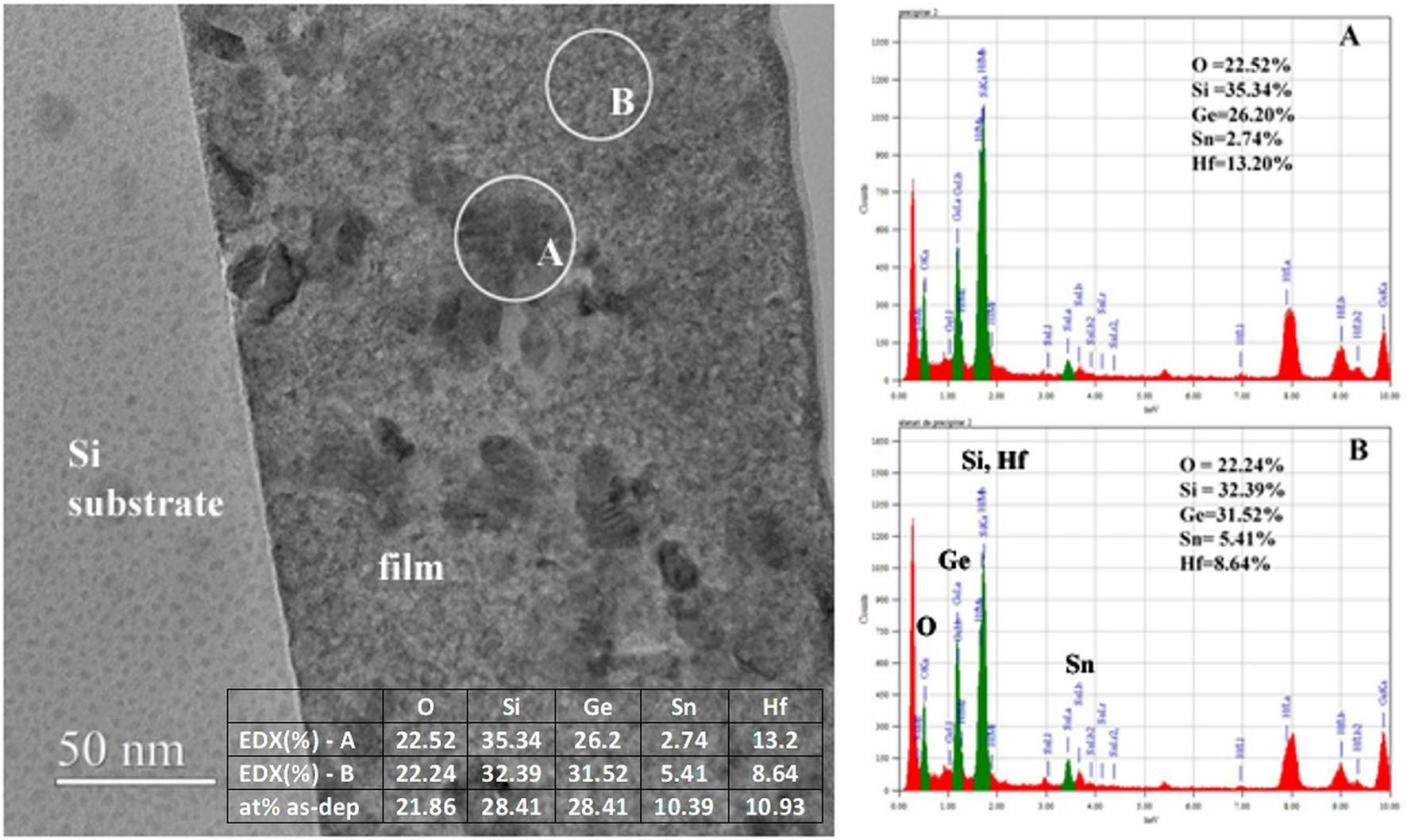
TEM image of the RTA 550 °C sample showing different contrast areas and corresponding EDX spectra for the marked regions A and B in the TEM image. The inset table shows the comparison of EDX concentrations with estimated at.% values for as-dep sample based on volume ratio composition.

By annealing at a higher temperature of 550 °C, the crystallization is extended to the whole volume of the film as illustrated in Figs. 1c and 3. The EDX spectrum acquired from a dark region labeled A in Fig. 3 reveals a higher Hf content and a low Sn concentration, while B region with less contrast reveals a smaller Hf content and higher Sn concentration, as exemplified by the insets A and B of EDX spectra in Fig. 3. The dark contrast of A dark region is due to the crystalline Bragg contrast of the HfO_2_ NCs component. The EDX concentrations of regions A and B are in reasonable agreement with the at.% values for as-dep sample estimated based on volume ratio composition given in the “Methods” section, as shown by the table inserted in Fig. 3. The value of Sn concentration obtained from volume ratios was found higher than the EDX values, perhaps due to overestimation of the thickness of Sn film used for calibration, because of a possible granular structure.

**Figure 4:**
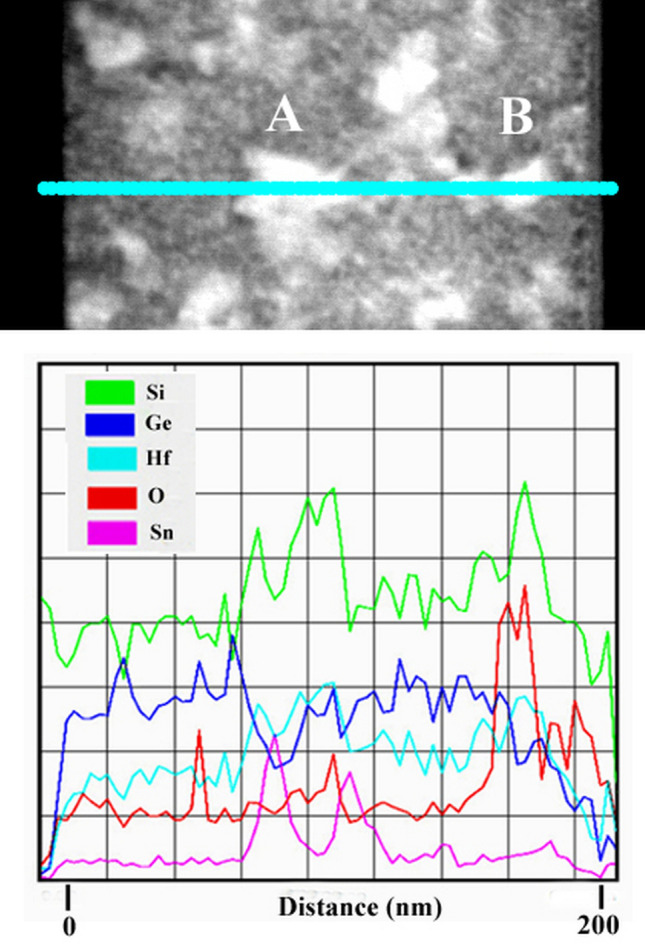
STEM-HAADF image and line scan spectrum for the section of the 550 °C sample.

Complementary information about the atoms segregation in the crystallization process in 550 °C sample is revealed by STEM –HAADF image along a line-scan analysis as shown in Fig. 4. Here, the crystals appearing white in Z contrast image (labeled as A and B in Fig. 4) have higher Hf atoms content. The white Z contrast can be also present in the high Sn content regions as revealed by the scan line of A particle in Fig. 4, where the central part is rich in Hf and Sn peaks appear laterally. However, for particle B, the presence of the Hf and O clearly indicates the formation of a HfO_2_ structure. This fact indicates a segregation of Hf into the crystallized areas of HfO_2_, accompanied by a displacement of SiGeSn outside these areas of HfO_2_ network, where SiGeSn NCs are nucleated.

**Figure 5:**
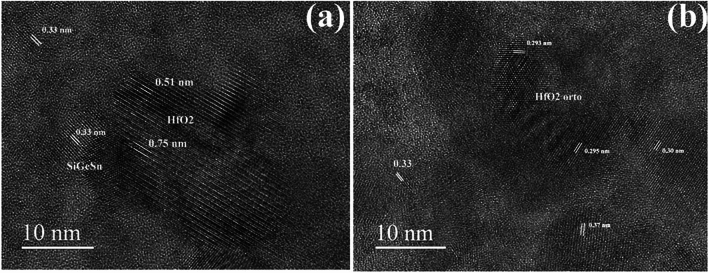
(**a**) HRTEM image of a black contrast crystallite showing a 25 nm HfO_2_ crystallite, in contact with a smaller (10 nm) crystallite of SiGeSn, in near [110] orientation; (**b**) HRTEM image of a HfO_2_ orthorhombic crystallite.

The HfO_2_ network is also evident in HRTEM images shown in Fig. 5. Similar to Fig. 3, the HfO_2_ network regions of large crystallites appear with black contrast. The analysis of these images and those of SAED patterns shows that the HfO_2_ network is mostly orthorhombic, but there is also the monoclinic phase as well as the intermediate phase with lower monoclinicity (deformation), which appears under stress conditions^30^. This is highlighted in Fig. 5b by the fringes with a periodicity of 0.300 nm (intermediate between 0.295 nm for orthorhombic and 0.313 nm for monoclinic).

To conclude, the cubic structure of the SiGeSn crystallites appears after annealing, all over the film, in the areas with less Hf. These crystallites have sizes between 5 and 10 nm are distributed in the volume of the film and are highlighted by the fringes with a periodicity of 0.330 nm. The analysis of TEM observations allows advancing the hypothesis that the HfO_2_ network nucleates first and grows relatively quickly through the segregation (diffusion) of Hf from the areas adjacent to the nucleation. At the same time, there is a slower uniform nucleation of the cubic structure of the SiGeSn alloy, which forms small crystallites, below 5 nm after the 500 °C RTA, and which grows more (up to 10 nm) after the 550 °C RTA. Some observations suggest that the HfO_2_ network, once nucleated, can serve as a support for the nucleation of the cubic SiGeSn phase, a process that could be eased by the diffusion of Hf necessary for the growth of the HfO_2_ network and the opposite diffusion of Ge and Sn in amorphous matrix.

#### XRD results

**Figure 6:**
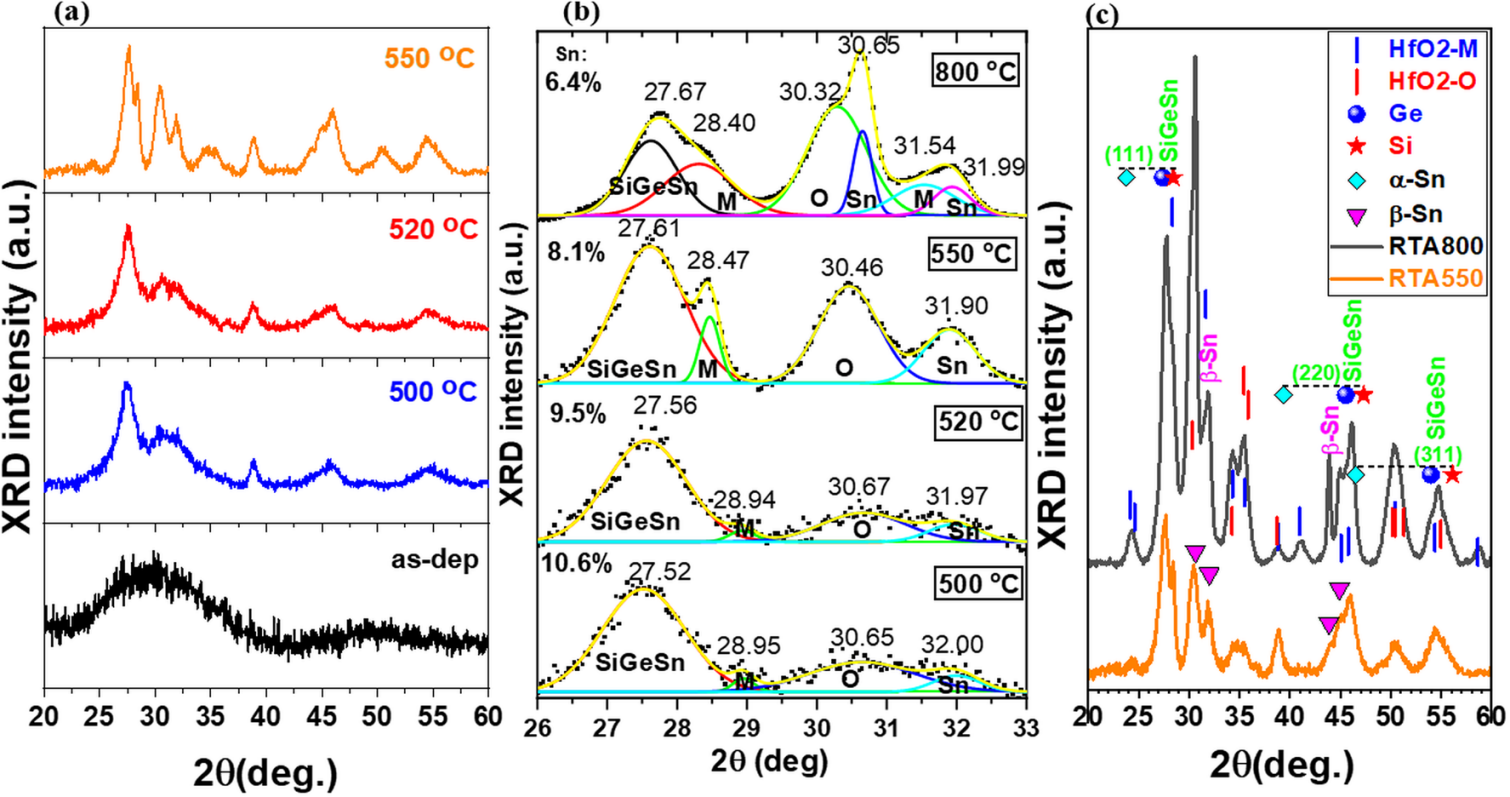
X-Ray diffractograms taken on SiGeSn-HfO_2_ structures annealed at 500, 520, 550 and 800  °C: (**a**) the as-dep film and films RTA treated at 500, 520 and 550  °C; (**b**) normalized curves and peaks from deconvolution for all RTA samples; (**c**) comparison between RTA 550  °C and RTA 800 °C samples and XRD positions shown as labels in figure (**c**) for Si (PDF No. 01-070-5680), Ge (PDF No. 04-013-4796), α-Sn (PDF No. 01-071-4637) and β-Sn (PDF No. 01-071-4637); HfO_2_ monoclinic M (PDF No. 01-078-0049) and orthorhombic O (PDF No. 00-021-0904). Labels in figure (**b**) are: SiGeSn, HfO_2_ M and O and Sn for β-Sn. The values of Sn/SiGeSn (%) content estimated based on (111) SiGeSn peak positions for different RTA temperatures are also given as labels in figure (**b**).

As can be imagined, the SiGeSn crystallization process is hindered if it is embedded in an amorphous oxide matrix, this process requiring the diffusion-segregation for NCs nucleation. Previous studies on a tri-layer memory structure based on a thin floating gate layer of SiGeSn embedded in HfO_2_^31^ have revealed that the annealing temperature required for the formation of SiGeSn NC is in the range of 500–550 °C. This is also valid for thick amorphous layers of SiGeSnHfO_2_ studied in this paper as resulted from HRTEM analysis presented above and the X-ray diffractograms in Fig. 6a for layers with different RTA temperatures. Better crystallization was obtained for the sample annealed at 550 °C with formation of both SiGeSn and HfO_2_ NCs (in agreement with HRTEM and Raman).

For identification of the diffraction peaks, the diffractograms with well-defined peaks of the RTA 550 °C and RTA 800 °C samples are compared in Fig. 6c, with the peak positions given in XRD database for Si, Ge, α- and β-Sn, monoclinic and orthorhombic HfO_2_. Thus, we can attribute the broad peak at 27.61°–27.67° (Fig. 6b) to NCs of SiGeSn being positioned between the lines corresponding to Si cubic (PDF: 01-070-5680), Ge (PDF No. 04-013-4796) and α-Sn cubic (PDF No. 01-071-4637) (Fig. 6c).

The SiGeSn NC diameter estimated from Scherrer's formula is about 8 nm. The RTA 500 °C sample contains SiGeSn NC with a higher concentration of Sn compared to the structure treated at 800 °C (through the lower 2θ position for 500 °C RTA, which corresponds to a higher lattice constant, an effect expected with the introduction of Sn), for the latter, highlighting the strong segregation of β-Sn can be seen in samples annealed at 550 °C and 800 °C (Fig. 6b). The values of Sn/SiGeSn (%) content in SiGeSn NCs for different RTA temperatures have been estimated by linear interpolation of (111) SiGeSn peak positions in respect to those of Si, Ge and α-Sn and are shown as labels in Fig. 6b. For that, we approximated SiGe in SiGeSn NCs as Si_0.5_Ge_0.5_ component (that of the sputtering target). We can see that Sn content decreases by increasing the RTA temperature due to Sn segregation from 10.6% for 500 °C to 6.4% for 800 °C. For RTA 800 °C, Sn/SiGeSn content in NCs evaluated by Raman scattering analysis presented below is 5%, in fair agreement with 6.4% obtained by XRD (Fig. 6b). For the sample RTA 550 °C, the XRD value of 8.1% for Sn/SiGeSn is close to 7.8% value computed based on the EDX composition of the spot B in Fig. 3.

In the RTA 550 °C sample, the monoclinic HfO_2_ peak (M) at 28.47° is clearly defined, while the broad peak at 30.46 could be mainly due to orthorhombic HfO_2_ phase (O) with contributions from segregated disordered β-Sn and monoclinic HfO_2_ peak. The monoclinic and orthorhombic HfO_2_ peaks are better resolved within the diffractogram of the RTA 800 °C sample. We increased the RTA temperature to 800 °C to see up to what temperature Sn segregation and formation of orthorhombic and monoclinic HfO_2_ NCs occur. From RTA 800 °C and 550 °C spectra on extended scale in Fig. 6c**,** we can also assign the peak at 44° to β-Sn and that at about 46° to (220) SiGeSn diffraction with a shoulder from β-Sn and HfO_2_. The peak at ~ 55° has contributions from HfO_2_ and (311) SiGeSn diffractions. The crystallization of the HfO_2_ matrix with a mixture of monoclinic and orthorhombic structures was also demonstrated by our previous studies on Ge NCs films in HfO_2_ annealed at 550 °C^32,33^. The maxima located at 24°, 34.3°, 35.5°, 38.7°, 41.0°, 50.4° in Fig. 6c correspond to HfO_2_ NCs.

To conclude, the XRD results are in good agreement with those from HRTEM and spectral Raman investigations.

#### Raman spectroscopy results

**Figure 7:**
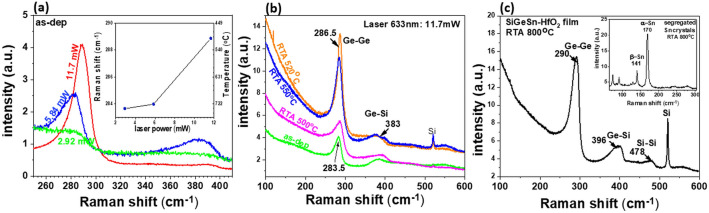
Raman spectra measured on samples SiGeSn-HfO_2_: (**a**) as-dep sample for different laser power excitations (the inset shows the laser power dependence of the Ge–Ge peak position and evaluated local temperature); (**b**) spectra acquired with maximum laser power excitation of 11.7 mW on as-dep sample in comparison to the samples after RTA at 500, 520, and 550 °C; (**c**) spectrum measured on sample annealed at 800 °C (inset is the spectrum measured on a segregated β-Sn crystal).

To assess the nanocrystallization level of the sample, we employed µ-Raman spectroscopy using an excitation 633 nm laser light. The Raman spectrum measured on as-dep sample for a low laser power (2.9 mW) corresponds to the amorphous SiGeSnHfO_2_ with emission in the region of wavenumbers smaller than 300 cm^−1^, due to the vibration of Ge–Ge and Ge–Sn bonds (Fig. 7a). By increasing the laser power to 5.8 mW, the laser exposure during the measurements results in local heating and nanocrystallization of the film with strong increase of the peak originating from Ge–Ge vibrations in SiGeSn NCs. Due to low Sn content in the as-dep SiGeSnHfO_2_ film, the contribution of Ge-Sn vibrations is detected only as an extension of the Ge–Ge peak to lower wavenumber. The Raman spectrum shows a broad peak at ~ 383 cm^−1^ due to Ge–Si vibrations, but no Si–Si mode, except that at 520 cm^−1^ from Si substrate, even if SiGe (50:50) target was used for sputtering deposition. This means that Si incorporation into NCs is quite low. To explain the low Si content in SiGeSn NCs, one should consider the diffusion and segregation of Ge and Sn atoms in HfO_2_ matrix is faster than for Si, during heating^7^.

For the maximum laser power of 11.7 mW, the Ge–Ge peak intensity is increased and shifted to higher wavenumber to 288.6 cm^−1^, closer to 300 cm^−1^ value for pure c-Ge, due to the increase of the NCs size and better nanocrystallization. The nanocrystallization level depends not only on the laser power, but also on the duration of the laser exposure. The laser power dependence of the Ge–Ge peak position and evaluated local temperature are shown in the inset of Fig. 7a. For evaluation of the local temperature, we have used the calibrated thermal coefficient η = 0.0156 cm^−1^ K^−1^ of the temperature dependence Ge–Ge peak *ω* = *ω*_*o*_*—ηΔT* which was shown to be almost independent on GeSn composition up to 14% Sn (*ω*_*o*_ the peak position for bulk Ge at room temperature*, ΔT* temperature variation)^34^. The peak positions depend also on the composition, size and strain of the SiGeSn NCs from where the Raman scattering occurs.

The nanocrystallization induced by laser light exposure is very local, restricted to the laser spot diameter of 1–2 µm, with high temperature gradients in-plane and cross-plane directions that influence the diffusion and segregation of atoms. Such Raman studies are interesting as they contribute to understanding the nanocrystallization processes, but they are significantly different than the results of the uniform entire film transformation obtained by RTA. Raman spectra measured on samples annealed by RTA at different temperatures (500, 520 and 550 °C) for laser power of 11.7 mW are shown in Fig. 7b in comparison to the spectrum of as-dep sample modified by the laser exposure during the measurement. Just to mention, up to maximum laser power, there is negligible effect of the laser exposure in case of annealed sample. We can see in Raman spectra (Fig. 7b) that the maxima observed on laser annealed samples and discussed above are also found on spectra of RTA samples: the Ge–Ge peak in the range 286.5 cm^−1^; the Ge–Si peak at about 383 cm^−1^, more pronounced in sample annealed at 550 °C. The RTA at higher temperatures than 600 °C results in a strong Sn diffusion and segregation at the film surface. The Raman spectrum measured on RTA 800 °C is presented in Fig. 7c. Beside the already discussed Ge–Ge (290 cm^−1^) and Ge-Si (396 cm^−1^) peaks, the Si–Si peak at 478 cm^−1^ is clearly detected, the sign that Si concentration in SiGeSn NCs is increased and Sn content is reduced. Using an empirical relation between the ratio of the Si–Ge and Ge–Ge peak areas (*R* = *I*_SiGe_/*I*_GeGe_ = 2(1 – *x*)/*Bx* with *B* = 1.5) and neglecting the Sn alloying influence, the Si concentration of 25% is evaluated^35^. From the Ge–Ge peak position as a function of Si (*x*) and Sn (*y*) concentrations (Ge–Ge [cm^−1^] = 300 – 19.2*x* – 93.5*y*) a small content of Sn less than 5% is roughly estimated^36^. We evaluated the Sn concentration in SiGeSn NCs only for RTA 800 °C sample where Si–Si peak is better detected. As also shown by XRD studies, a strong β-Sn segregation occurs at RTA temperature of 800 °C, and Sn/SiGeSn content estimated by XRD (Fig. 6b) is close to that of 5% from Raman measurement. We have also detected by optical microscopy Sn segregation by formation of Sn crystals on the film surface. The µ-Raman spectrum measured on this kind of segregated crystal shows sharp peaks that can be assigned to a mixture of β-Sn and α-Sn phases in Sn crystals with Ge-rich regions^37^.

We conclude that the Raman scattering results agree with those of HRTEM and XRD, and add some additional knowledge about the crystallization process. Also, to note that, in comparison to previous studies on GeSn NCs in SiO_2_ (nanocrystallization in the range of 300–450 °C), the formation of SiGeSn NCs in HfO_2_ occurs at higher temperatures 500–550 °C due to better thermal stability induced by Si alloying.

#### Spectral photosensitivity

**Figure 8:**
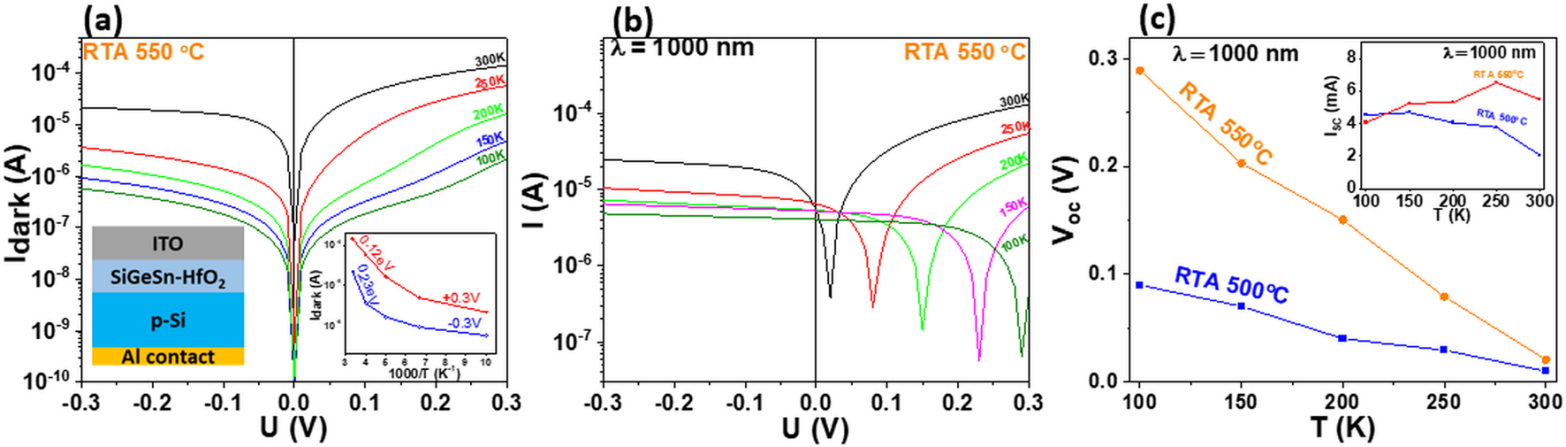
*I*–*V* dc measurements on ITO/SiGeSn-HfO_2_/p-Si/Al diodes at different measurement temperatures: (**a**) dark current I_dark_– V curves of RTA 550 °C sample (insets are schematic of the diodes structure and the temperature dependence of the dark current for direct (+ 0.3 V) and indirect (−0.3 V) bias voltages); (**b**) *I*–*V* curves of RTA 550 °C sample under constant monochromatic light of 1000 nm wavelength, ~ 0.3 mW/cm^2^; (**c**) temperature dependence of zero- current voltage V_oc_ for 500 °C and 550 °C RTA samples measured under 1000 nm wavelength illumination (the inset shows the corresponding short-circuit I_sc_ function of measurement temperature). For all figures, the current is in absolute values.

The *I–V* current–voltage measurements are performed on ITO/SiGeSn-HfO_2_/p-Si/Al diodes with films of SiGeSn NCs in HfO_2_ matrix annealed at different RTA temperatures, in dark and under light. The dark current *I–V* curves measured in 100–300 K temperature range on sample RTA 550 °C are illustrated in Fig. 8a. Similar results are obtained on samples with the active layers annealed by RTA at 500 °C (see Supplementary Information (SI) Fig. S1). A weak rectifying character with only about one order of magnitude higher “direct” current at + 0.3 V than the “indirect” one at − 0.3 V for all measurement temperatures is evidenced. The activation energy of these direct and indirect currents shows near room temperature values of 0.12 eV and 0.23 eV, respectively, and strongly decreases at low temperatures when the current is controlled by tunneling (inset of Fig. 8a).

Figure 8b shows the *I*–*V* curves of the total current (dark + photocurrent) measured at different temperatures on RTA 550 °C sample under 1000 nm wavelength light. The photovoltaic effect is revealed by the positive open circuit (zero current) voltage *V*_oc_ which increases from 0.025 to 0.3 V as the temperature decreases from 300 to 100 K. The *V*_oc_ temperature dependence of the RTA 550 °C sample is compared in Fig. 8c with that of the RTA 500 °C sample. The positive *V*_oc_ voltage agrees with the rectifying behavior of the diode in which the direct current corresponds to the positive voltage (inset of Fig. 8a). The temperature dependence of the corresponding short circuit current *I*_sc_ for *λ* = 1000 nm illumination is also shown for both investigated diodes by the inset in Fig. 8c. As expected, *I*_sc_ is almost the same for the two diodes and is slightly dependent on temperature, and has smaller value at room temperature in case of the RTA 500 °C, due to higher photocarrier recombination in this SiGeSn-HfO_2_ layer still containing amorphous phase (see HRTEM section). This is accompanied by smaller *V*_oc_ values found at this RTA 500 °C diode (Fig. 8c and Fig. S1 in SI).

**Figure 9:**
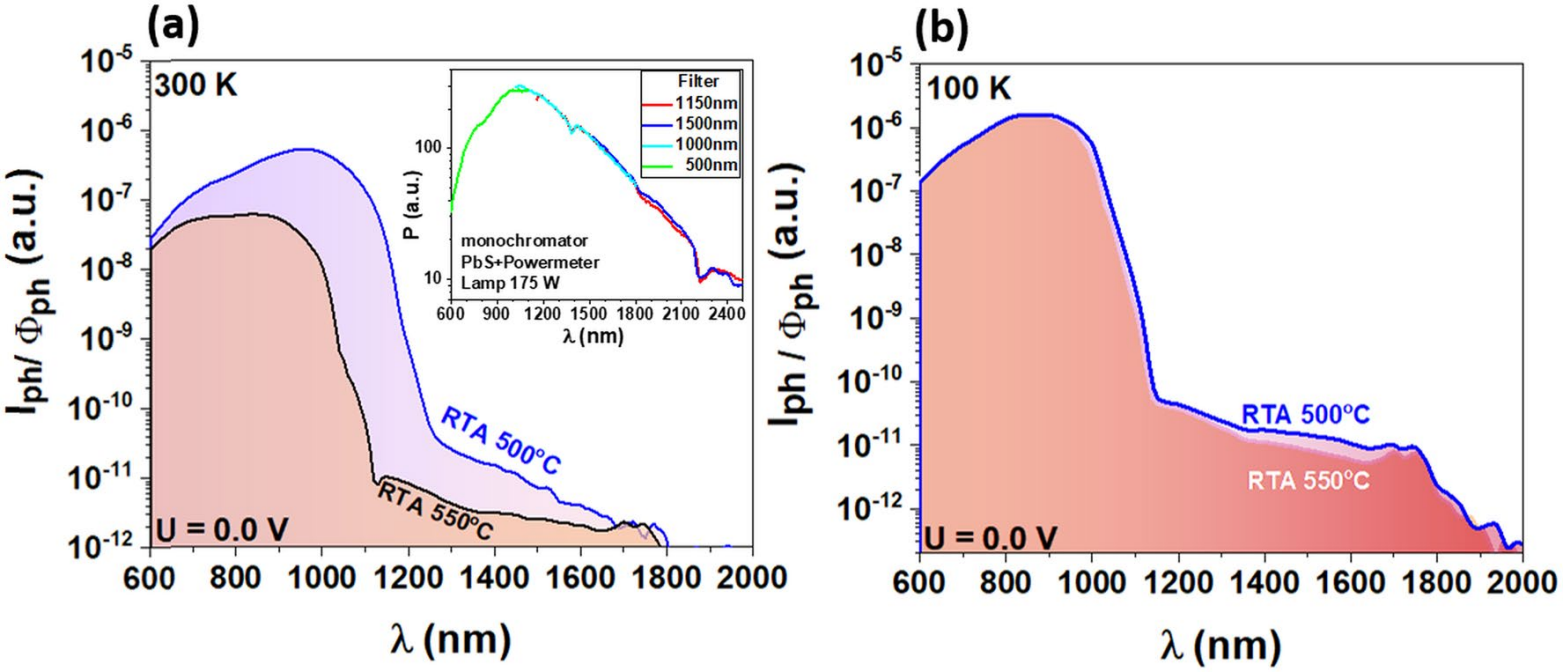
Spectral ac photosensitivity measured for zero bias voltage on ITO/SiGeSn-HfO_2_/p-Si/Al diodes, for RTA at 500 and 550 °C at: (**a**) 300 K and (**b**) 100 K. The calibrated *Φ*_ph_ spectrum is shown by the inset of figure (**a**).

The spectral sensitivity of the ITO/SiGeSn-HfO_2_/p-Si/Al diodes for 0 V applied voltage (short-circuit) and chopped monochromatic light in the wavelength range of 600–2000 nm was measured by using lock-in technique and shown in Fig. 9 for both RTA 500 °C and RTA 550 °C samples. The spectral photocurrent *I*_*ph*_ induced by the internal electric field of the heterojunction diode (photovoltaic current) was normalized to the spectrum of the monochromatic light *Φ*_ph_ and represented in Fig. 9a, b for 300 K and 100 K measurement temperatures. As can be seen, SWIR photodetection wavelength limits are almost the same for both diodes, i.e. 1800 nm at 300 K (Fig. 9a) and 2000 nm at 100 K (Fig. 9b). The weak decrease of the photosensitivity from low to room temperature, in contrast to previous results on GeSn NCs in SiO_2_, can be explained by a better NCs passivation induced by HfO_2_ matrix. Also, as shown in the Introduction section, the increased thermal stability by adding Si to GeSn allows a higher nanocrystallization temperature without β-Sn segregation, that results in the formation of better crystalline quality NCs, increasing the photosensitivity.

As discussed above, the photovoltaic current is mainly given by the photocarriers generated in the region of the internal electric field at the heterojunction interface of SiGeSn NCs with p-Si substrate. There are two distinct regions of the photosensitivity spectra, defined by the bandgap difference between Si substrate and SiGeSn NCs. Thus, in SWIR region the photocarriers are generated in SiGeSn NCs of smaller band gap, while for the VIS-IR region up to about 1200 nm, the main contribution to the photocurrent is given by the photoabsorption in Si crystalline substrate. The photosensitivity limit of ~ 1800 nm at room temperature and of ~ 2000 nm are related to the bandgap of the SiGeSn NCs in which the bandgap increases due to NC confinement effect^38^ is compensated by Sn alloying^39^. It should be noted that these SWIR sensitivity limits are within the sensitivity limit of our equipment and measurement conditions. An important factor is the spectral dependence of the spectral light intensity that is shown as inset of the Fig. 9a. For comparison between samples, same measurement conditions have been used.

The electronic transport mechanism in active layer with separated SiGeSn NCs in HfO_2_, consists in tunneling through passivation thin layer between neighbor NCs, or by thermally activated hoping for lower NCs concentration. It is important to optimize the NCs concentration for a balance between high electrical conductivity at high NCs concentration, on one hand and HfO_2_ induced good passivation for lower NCs concentration, on the other hand. This results in the formation of conductive paths between electrodes, as a percolation process.

We have to note comparing the spectra in Fig. 9a, b for the range of 1200–1800 nm, the photosensitivity is lower at high temperatures as a result of increased recombination rate of the photocarriers at high temperatures, especially in case of the RTA 500 ^o^C sample. More pregnant decrease of the photosensitivity at higher than 200 °C temperatures was observed in case of GeSn NCs immersed in SiO_2_ as reported in Ref.^7^. The results suggest a better passivation effect induced by the HfO_2_ matrix and a higher thermal stabilization of SiGeSn NCs by alloying with Si.

Also, interesting to note is that the RTA 550 ^o^C spectrum at room temperature shows smaller sensitivity than that of RTA 500 ^o^C sample (Fig. 9a), while the DC measurements of the I_sc_ for 1000 nm constant illumination is even slightly higher (inset in Fig. 8c). This could be explained by different influence of photocarrier trapping in DC and AC measurements.

## Conclusions

Amorphous layers of SiGeSnHfO_2_ were obtained by MS co-deposition using separate targets of SiGe, Sn and HfO_2_ targets. SiGeSn NCs in HfO_2_ matrix have been obtained by post deposition RTA at different temperatures. Best results, with SiGeSn NCs and no Sn segregation, were obtained for RTA temperature 500 °C. The RTA 550 °C sample shows also the formation of HfO_2_ NCs and the SiGeSn NCs closed HfO_2_ crystals edges. Strong segregation of β-Sn is evidenced for RTA at 800 °C by XRD and Raman investigations. Photovoltaic current at zero voltage bias has been measured on ITO/SiGeSn-HfO_2_/p-Si/Al heterojunction diodes for different RTA states of the active SiGeSn-HfO_2_ layer and different measurement temperatures, showing the spectral sensitivity extended at low temperature of 100 K to 2.0 µm in SWIR and limited to 1.8 µm at room temperature. In comparison to previous results on GeSn NCs in SiO_2_ matrix, the layers of SiGeSn NCs in HfO_2_ offer a better thermal stability and NCs passivation due to Si alloying and HfO_2_ matrix, respectively.

## Methods

### Preparation of SiGeSn NCs embedded in HfO_2_

SiGeSnHfO_2_ films were deposited on p-Si substrates (7–14 Ωcm resistivity) at room temperature by co-sputtering from three independent targets: GeSi (50:50 at.% atomic ratio) and Sn in DC sputtering regime, while HfO_2_ by RF. For each target, the plasma power was adjusted so that the obtained GeSi:HfO_2_:Sn ratio is 64:21:15 vol.% as estimated from calibrated deposition rates. From volume ratios, at. % composition of as-dep film was found to be: O:21.86%; Si:28.41%; Ge:28.41%; Sn:10.39%; Hf:10.93% and compared with EDX results in Fig. 3. Sputtering atmosphere was maintained at 4 mTorr under 25 sccm Ar 6N flow. The as-dep films have about 250 nm thickness. Rapid thermal annealing (RTA) at 500, 520 and 550 °C respectively was performed in Ar 6N for SiGeSn NCs formation.

### Measurement methods

The samples of SiGeSn:HfO_2_ were structurally and morphologically characterized by high resolution transmission electron microscopy (HRTEM), X-ray diffraction (XRD) and Raman spectroscopy measurements to determine the influence of manufacturing technological parameters on the formation of nanostructures.

The Sn concentration was also evaluated from HRTEM measurements of the lattice spacing of SiGeSn (111) planes and from selected area electron diffraction (SAED) patterns. The XRD measurements were performed using Rigaku SmartLab X-ray diffraction equipment, which uses monochromatic Cu K_α1_ radiation (λ = 1.542 Å). Raman scattering was performed by using a high resolution μ-Raman spectrometer (LabRAM HR800 Horiba) with laser excitation wavelength of 633 nm.

SiGeSn in HfO_2_ nanocrystallized films deposited on p-Si substrates have been characterized by electrical and photoelectrical measurements using ITO/NC SiGeSn -HfO_2_/p-Si/Al diodes (0.1 cm^2^) with a conductive transparent ITO and Al layers as top and bottom electrodes, respectively (see insert in Fig. 9a). ITO layers were deposited by RF sputtering on Si substrate heated at about 200 °C, while Al was thermally evaporated in vacuum. The measurements of electrical and photoelectrical properties were carried out in an optical cryostat, in a "sandwich" geometry. The photoelectric measurements were performed by illumination with modulated monochromatic light (frequency of 119 Hz) in the wavelength range from 600 to 2000 nm, at different sample temperatures in AC photovoltaic regime (external applied voltage U = 0 V), using a Stanford Research SR 810 lock-in amplifier and a Cornerstone 260 monochromator with 550 nm, 1000 nm and 1500 nm longwave pass filters. The photocurrent spectra were obtained by normalizing the measured photocurrent to the spectrum of the lamp. Dark and under monochromatic light DC currents were measured by using a Keithley 236 Source Measure Unit.

### Supplementary Information

Supplementary Information.

## Data Availability

All data generated or analysed during this study are included in this published article [and its supplementary information files].
