# Supplementary material for: Enhancing SiGeSn nanocrystals SWIR photosensing by high passivation in nanocrystalline HfO2 matrix
Source: Sci Rep. 2024 Feb 12;14:3532. doi: 10.1038/s41598-024-53845-z (PMC10861535; doi:10.1038/s41598-024-53845-z)
Supplement: Supplementary file 1 — Supplementary Information. [file 41598_2024_53845_MOESM1_ESM.pdf]

Enhancing SiGeSn nanocrystals SWIR photosensing by high passivation in nanocrystalline HfO_2 matrix

Supplementary Information

Ioana Dascalescu [1], Catalin Palade [1], Adrian Slav [1], Ionel Stavarache [1], Ovidiu Cojocaru [1], Valentin Serban Teodorescu [1,2], Valentin-Adrian Maraloiu [1], Ana-Maria Lepadatu [1,*], Magdalena Lidia Ciurea [1,2,*] and Toma Stoica [1,*]

[1] National Institute of Materials Physics, 405A Atomistilor Street, 077125 Magurele, Romania

[2] Academy of Romanian Scientists, 54 Splaiul Independentei, 050094 Bucharest, Romania

[*] Corresponding authors: Dr. Toma Stoica – toma.stoica@infim.ro, Dr. Ana-Maria Lepadatu – lepadatu@infim.ro, Dr. M. L. Ciurea – ciurea@infim.ro

The I-V current-voltage measurements are performed on ITO/SiGeSn-HfO_2/p-Si/Al diodes with SiGeSn NCs in HfO_2 matrix, in dark, for SiGeSn-HfO_2 layers annealed at RTA 500 °C. The dark current I-V curves measured in 100 – 300 K temperature range on sample RTA 500 °C are illustrated in **Fig. S1**, the dependence of the dark current on the voltage is almost symmetrical.

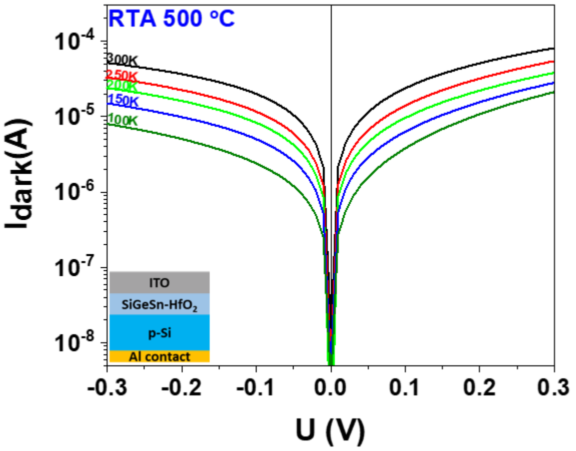

Fig. S1: I-V dc measurements on ITO/SiGeSn-HfO_2/p-Si/Al diodes at different measurement temperatures: dark current I_{dark}- V curves of RTA 500 °C sample
